# Fully Synthetic Longitudinal Real-World Data From Hearing Aid Wearers for Public Health Policy Modeling

**DOI:** 10.3389/fnins.2019.00850

**Published:** 2019-08-13

**Authors:** Jeppe H. Christensen, Niels H. Pontoppidan, Rikke Rossing, Marco Anisetti, Doris-Eva Bamiou, George Spanoudakis, Louisa Murdin, Thanos Bibas, Dimitris Kikidiks, Nikos Dimakopoulos, Giorgos Giotis, Apostolos Ecomomou

**Affiliations:** ^1^Eriksholm Research Centre, Oticon A/S, Snekkersten, Denmark; ^2^Department of Computer Science, University of Milan, Milan, Italy; ^3^The Ear Institute, Brain Institute, UCL, London, United Kingdom; ^4^Department of Computer Science, City University of London, London, United Kingdom; ^5^Guy's and St. Thomas' NHS Foundation Trust, London, United Kingdom; ^6^Department of Otolaryngology, National & Kapodistrian University of Athens, Athens, Greece; ^7^ATC Innovation Lab, Athens, Greece; ^8^Athens Medical Group, Athens, Greece

**Keywords:** real-world data, longitudinal data, hearing aids, public health policy, evidence-based, hearing loss, sound exposure

## Introduction

Approximately one-third of people over 65 years of age, and 5% of the world's population, is affected by hearing loss (HL) (World Health Organization, 2017). Disabling HL is associated with early cognitive decline in adults (Olusanya et al., 2014), and when unaddressed, HL restricts social integration and reduces employment and educational opportunities, hampers emotional well-being and, thus, poses an economic challenge at both the individual and national level (Wilson et al., 2017). Moreover, more and more individuals suffer from HL, which is primarily due to increases in everyday noise exposure and an increase of the aging population (World Health Organization, 2017). Despite the fact that age-related HL is the third leading cause of years lived with disability (Vos et al., 2017), the population of individuals with hearing loss is underserved because few public health policies focus on prevention, intervention, and rehabilitation for age-related HL (Reavis et al., 2016). This inadequate focus has been attributed to a lack of evidence supporting policies that actively promote hearing healthcare (Moyer, 2012; Barker et al., 2016). However, this specific issue is targeted in the EU-funded H2020 project EVOTION (www.h2020evotion.eu), which collects a large volume of heterogenous data from almost 1,000 hearing aid (HA) users with varying degrees of hearing loss to support the development of evidence-based policy making within the hearing healthcare field (Spanoudakis et al., 2017; Gutenberg et al., 2018). Data are being collected from five sources: (i) hearing aids, (ii) a smartphone app, (iii) a biosensor, (iv) audiology clinics, and (v) electronic health records. The hearing aids log data about the user's sound environment (Pontoppidan et al., 2018), hearing aid use (i.e., on/off) and hearing aid settings on a minute-by-minute basis; a phone app developed for the study collects information about the user's physical location via GPS (Dritsakis et al., 2018). Thus, EVOTION will provide an evidence base for formulating and evaluating the impacts of public health policy pertaining to prevention, early diagnosis, and treatment/rehabilitation for adults with hearing impairment.

Here, we share the first outcome of EVOTION in the form of a data-set to inspire, encourage, and motivate a data-driven analytical approach to evidence-based healthcare policy modeling using real-world longitudinal data. The data-set includes information relating to patterns of real-world hearing aid usage and sound environment exposure. Undoubtedly, many such data-sources will be available for researchers and policy-makers in the future, and the data-set presented here can act as a first step of building and testing potential statistical models (Christensen et al., 2018, 2019).

Specifically, the data-set represents a sub-sample of the data being collected in EVOTION. It contains longitudinally sampled observations from 53 individuals and includes the following measures: the sound environment, the hearing aid setting, logging time (timestamps), ID, and the degree of hearing loss on the best hearing ear of the individuals. Note that the ID (an integer between 1 and 53, randomly assigned to each individual) does not link to the real identity of the participants.

Data are considered sensitive as they contain personal and health related information, and EVOTION adhere to strict data ethics by applying privacy-aware big data analytics (Anisetti et al., 2018). Here, we overcome the problem of sharing such personal data by working with a fully synthetic data-set that preserves structural and statistical properties of the original data (see section Technical Validation), without allowing the extraction of personal information (see section Data Synthesization). Thus, the synthetic data-set can readily be shared among professionals.

## Methods

### Protocol

Data collection in EVOTION follow a published protocol (Spanoudakis et al., 2017; Dritsakis et al., 2018), is ongoing, and spans 12 months from the day of recruitment to the end of study participation. The data-set presented here represents a synthesized data-sample from EVOTION. The source data span a mean of 17 days of hearing aid usage (minimum 2 and maximum 54 days), 53 participants, and a total of ~5,000 h of hearing aid usage.

### Data Acquisition

Each participant in EVOTION is supplied with a pair of EVOTION hearing aids and a Samsung A3 smartphone. The hearing aids are connected to the smartphone via low-energy Bluetooth, and a custom developed EVOTION app (developed by ATC, Athens) on the smartphone logs a real-time data vector every minute consisting of data parameters from both the hearing aid's processing of sound from the microphone, hearing aid settings, and the smartphone's GPS. When connected to a wireless network, the smartphone app transmits the logged data vector (see Table 1) to the EVOTION data repository, which is located on secure distributed servers.

**Table 1:** Data-set variables logged every minute.

**Variable name**	**Description**	**Type**	**Units/levels**
ID	Identifier	Integer	1:53
SoundClass	A value describing the sound environment. The value is derived by the hearing aids internal processing of the acoustic variables	Categorical	QUIET, SPEECH, SPEECH-IN-NOISE, NOISE
hProg	A value describing the active hearing aid program (Dritsakis et al., 2018)	Categorical	MEDIUM, LOW, HIGH, HIGH+
hVol	A value describing the active hearing aid volume state	Integer	Steps (−9:4) represent 2.5 dB up or down from default (0)
LonRel	Relative longitude (centered for each individual)	Continuous	GPS
LatRel	Relative latitude (centered for each individual)	Continuous	GPS
LowSPL	The sound pressure level (SPL) measured in low frequency bands	Continuous	dB
MidSPL	SPL measured in middle frequency bands	Continuous	dB
HighSPL	SPL measured in high frequency bands	Continuous	dB
fbSPL	SPL measured in full bandwidth	Continuous	dB
LowNf	The noise floor (Nf) measured in low frequency bands	Continuous	dB
MidNf	Nf measured in middle frequency bands	Continuous	dB
HighNf	Nf measured in high frequency bands	Continuous	dB
fbNf	Nf measured in full bandwidth	Continuous	dB
LowME	The modulation envelope (ME) measured in low frequency bands	Continuous	dB
MidME	ME measured in middle frequency bands	Continuous	dB
HighME	ME measured in high frequency bands	Continuous	dB
fbME	ME measured in full bandwidth	Continuous	dB
Timestamp	Local time of record	ISO 8601	YYYY:MM:DD HH:MM:SS
LowSNR	The signal-to-noise ratio (SNR) from low frequency bands as SNR = SPL – Nf	Continuous	dB
MidSNR	SNR from middle frequency bands	Continuous	dB
HighSNR	SNR from high frequency bands	Continuous	dB
fbSNR	SNR from full bandwidth	Continuous	dB
LowMI	The modulation index (MI) from low frequency bands as MI = ME – Nf	Continuous	dB
MidMI	MI from middle frequency bands	Continuous	dB
HighMI	MI from high frequency bands	Continuous	dB
fbMI	MI from full bandwidth	Continuous	dB
PTA4	Pure tone average (PTA) across 4 testing frequencies (0.5, 1, 2, and 4 kHz) on the best hearing ear	Continuous	dB hearing threshold

*Frequency bands corresponds to 0–1.33; 1.33–2.14; 4.14–10; and 0–10 kHz*.

Clinical (e.g., audiometric tests) and demographical data are collected from the hearing clinics that have been involved in recruiting participants for EVOTION.

### Acquisition of Acoustic Variables

The EVOTION hearing aids implements proprietary algorithms for continuous estimates of the acoustic environment sensed by the calibrated hearing aid microphones. The continuous estimates are derived by level estimators that implements very short time constants in four frequency channels: full bandwidth, low, mid, and high frequencies. The dynamic range of the estimators covers the dynamic range of the microphones. Noise floor is estimated from the lowest values and the modulation envelope from the largest values within a longer time-window. The SNR is obtained by subtracting the noise floor from the SPL, and the modulation index by subtracting the noise floor from the modulation envelope. Also, from the full bandwidth signal, a proprietary algorithm estimates if the current sound environment is quiet, noise, speech, or speech-in-noise dominated (i.e., the “SoundClass” variable in Table 1). In total, the acoustic environment is described by 21 variables, which the hearing aid transmits to the smartphone over Bluetooth every minute.

### Data Synthetization

To enable data-sharing, and uphold differential privacy, we synthesized the data-set from a subset of the EVOTION repository data (the source) using DataSynthesizer (for details, see Ping et al., 2017). First, DataSynthesizer generates empirical conditional probability density functions (PDFs) for each variable of the data-source by computing a Bayesian network using the GreedyBayes algorithm with up to 4 parents—that is, the values in one variable can be conditioned on the values of up to four other data variables. Next, the synthesized data-set is generated by randomly drawing from the empirical PDFs while injecting each drawn sample with Laplacian noise with location 0 and scale 4(*d* – *k*)/*n*ε to preserve privacy. Here, *n* is the size of the source input (rows), ε = 0.1, *d* is the number of variables, and *k* = 4. Thus, the covariance between source parameters are preserved by allowing the empirical PDFs to be conditioned in the Bayesian network. In addition, to mask absolute position from GPS measures, each latitude and longitude coordinate were centered for each individual (i.e., subtracted by the mean latitude and longitude) prior to synthesization.

### Limitations and Updates

While EVOTION collects a large amount of heterogeneous data, the dataset described here represents a sub-collection of the parameters from a sub-population of all the individuals enrolled in EVOTION, which limits the data-set's usability for hypothesis testing. We expect to update the data-set with more observations and data-types once these become available in the EVOTION project for synthesization. In addition, we do not have access to low-level details of the signal-processing taking place in the hearing aids. Thus, we do not include real-time data on how the hearing aids autonomously reacts to the sound environment (e.g., adjusting noise reduction or compression characteristics).

### Data Format

The data-set is stored as a comma separated values (csv) file with each row representing one vector of observations associated to a timestamp. The included 399.500 observations represent 28 variables (columns) and they are described in Table 1.

### Data Access

The newest version of the dataset is named “EVOreal_time_synth.csv” and is uploaded to zenodo.org and accessible via the following DOI: https://doi.org/10.5281/zenodo.2668210.

## Technical Validation

**Figure 1:**
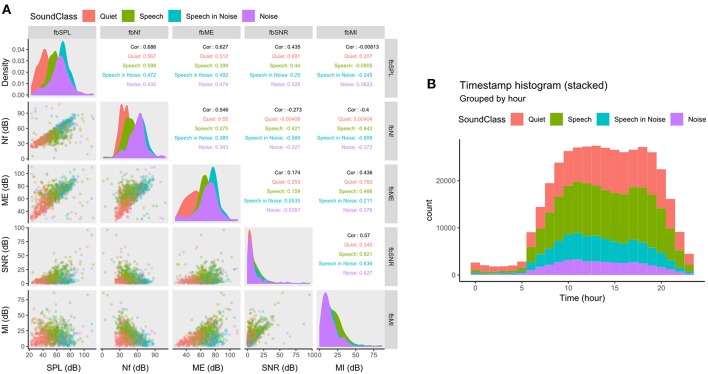
Scatterplot matrix (panels below the diagonal), density plots (panels in the diagonal), and correlation matrix (panels above the diagonal) of the full-bandwidth acoustic variables **(A)** and a stacked histogram of the “Timestamp” variable **(B)**. Data in both **(A,B)** are grouped and colored by the classified sound environment by the variable “SoundClass.” SPL, Sound pressure level; Nf, Noise floor; ME, Modulation envelope; SNR, Signal-to-noise ratio; MI, Modulation index.

The Bayesian network generating the fully synthetic data ensures that covariance between different variables are preserved. To validate that, indeed, dependencies are still present in the fully synthetic data-set we computed statistics from both the acoustic variables (only the full-bandwidth variables were selected) and the “Timestamp” variable (see Figure 1).

### Acoustic Variables

According to the hearing aids' estimation of the acoustic variables (see section Acquisition of Acoustic Variables), we would expect certain dependencies in the synthesized data. For example, the estimated noise floor (fbNf) should ideally always be lower than the estimated modulation envelope (fbME). The correlation matrix of the full bandwidth acoustic variables and their classification into the four discrete environments by color (“SoundClass”) are shown in Figure 1A. As expected, the noise floor is almost always lower than the modulation envelope (expect for a few outliers, see row 3, column 2 in Figure 1A). The outliers that do not follow the expected pattern are not generated by the synthesization process but instead reflects noise in the hearing aids' estimation method (outliers are also present in the source data). The color-coding indicates that clustering of the sound environment depends on more than one acoustic parameter. For example, in Figure 1A (panel in row 3, column 2), the environment is dominantly classified as “Quiet” for low levels of noise floor and modulation envelope. But as the modulation envelope passes ~60 dB the environment changes to either “Speech” or “Speech in Noise” despite no changes in the noise floor. This 3rd order dependency further validates that the data synthesization process preserves structural dependencies in the source data.

### Timestamps

Each observation in the data-set is associated with a timestamp. Thus, we can aggregate the timestamps to test the hypothesis that hearing aid usage is not uniformly distributed throughout the day and, from this, validate that the data synthesization process preserves the distributional statistics of the “Timestamp” variable. Figure 1B shows the histogram of timestamps binned by hour from 0 to 23. Most timestamps fall between 5 a.m. and 9 p.m. (usual awake hours) with peaks around noon and evening (6 p.m.). In addition, most data are logged in “Quiet” or “Speech” environments, which reflects what is reported in the literature (Humes et al., 2018). Thus, the distribution of timestamps and the dependency between timestamps and the sound environment both exhibit characteristics expected from real life use of hearing aids.

## Conclusion

We present a synthesized data-set containing longitudinal observations of hearing aid use and associated sound environments. The data represent real life behavior of individuals with hearing loss wearing hearing aids. The underlying reason for sharing these data is to motivate the use of such data for public health policy modeling—that is, identifying which models derive useful “high-level” information and insights from such “low-level” data observations in the field of hearing healthcare.

## Data Availability

All datasets generated for this study are included in the manuscript/supplementary files.

## Author Contributions

JC wrote the text, analyzed and synthesized the data-set, and prepared the figures. NP co-authored the text. RR, D-EB, LM, TB, DK, and AE recruited the participants. MA, GS, ND, and GG fascilitated the datalogging by technical developments.

### Conflict of Interest Statement

The authors declare that the research was conducted in the absence of any commercial or financial relationships that could be construed as a potential conflict of interest.
